# *Arabidopsis* cryptochrome is responsive to Radiofrequency (RF) electromagnetic fields

**DOI:** 10.1038/s41598-020-67165-5

**Published:** 2020-07-09

**Authors:** Maria Albaqami, Merfat Hammad, Marootpong Pooam, Maria Procopio, Mahyar Sameti, Thorsten Ritz, Margaret Ahmad, Carlos F. Martino

**Affiliations:** 1 Sorbonne Universités – UPMC Paris 6 – CNRS, UMR8256 - IBPS, Photobiology Research Group, 7 Quai St. Bernard, 75005 Paris, France; 2 Department of Biomedical and Chemical Engineering and Science, Florida Institute of Technology, 150W University Blvd, Melbourne, Fl 32901 USA; 3 Department of Biophysics, Johns Hopkins University, 3400N. Charles Street, Baltimore, MD 21218 USA; 4 Department of Physics and Astronomy, University of California at Irvine, Irvine, CA 92697 USA; 5 Xavier University, 3800 Victory Parkway, Cincinnati, Ohio, 45207 USA

**Keywords:** Biological sciences, Biochemistry

## Abstract

How living systems respond to weak electromagnetic fields represents one of the major unsolved challenges in sensory biology. Recent evidence has implicated cryptochrome, an evolutionarily conserved flavoprotein receptor, in magnetic field responses of organisms ranging from plants to migratory birds. However, whether cryptochromes fulfill the criteria to function as biological magnetosensors remains to be established. Currently, theoretical predictions on the underlying mechanism of chemical magnetoreception have been supported by experimental observations that exposure to radiofrequency (RF) in the MHz range disrupt bird orientation and mammalian cellular respiration. Here we show that, in keeping with certain quantum physical hypotheses, a weak 7 MHz radiofrequency magnetic field significantly reduces the biological responsivity to blue light of the cryptochrome receptor cry1 in *Arabidopsis* seedlings. Using an *in vivo* phosphorylation assay that specifically detects activated cryptochrome, we demonstrate that RF exposure reduces conformational changes associated with biological activity. RF exposure furthermore alters cryptochrome-dependent plant growth responses and gene expression to a degree consistent with theoretical predictions. To our knowledge this represents the first demonstration of a biological receptor responding to RF exposure, providing important new implications for magnetosensing as well as possible future applications in biotechnology and medicine.

## Introduction

Static magnetic fields have profound and diverse effects on living organisms ranging from prokaryotes to man^1–9^. One of the best characterized involves orientation behaviour in migratory birds, which use the magnetic field for directional sensing by a process that requires light^1,7^. Bird magnetosensing has been proposed to occur by the so-called radical pair mechanism, whereby weak magnetic fields can alter the spin characteristics of radical pairs generated by a biological magnetoreceptor^10^. An evolutionarily conserved flavoprotein photoreceptor known as cryptochrome, which forms radical pairs and is localized to the bird retina^1,11,12^, has been proposed as such a possible magnetoreceptor.

An intriguing feature of the radical pair hypothesis in birds is the theoretical prediction that RF signals in the 1–10 MHz range should elicit the disruption of bird directional responses to the Earth’s magnetic field^13–15^. Such disruptive effects were indeed found experimentally for RF fields, remarkably even of intensities below 10 nT^9^ and, in the case of broad-band fields, below 1 nT^16^. Many of these effects can in principle be rationalized with the radical-pair mechanism^14,17^. Therefore since cryptochromes have been implicated in responses to static magnetic fields in organisms ranging from plants to humans, a prediction of the radical pair hypothesis is that RF magnetic fields could also affect cryptochrome responses. In this work, we explored the intriguing possibility that such fields could trigger a biological response involving *Arabidopsis* cryptochrome

## Results

A rapid, quantitative, and direct assay for magnetic sensitivity is the *in vivo* phosphorylation of *Arabidopsis* cryptochrome in blue light^10,18^. Phosphorylation results from conformational changes triggered in the receptor, whereby the cryptochrome C-terminal domain unfolds from the protein surface and becomes accessible to cellular kinases. Cryptochrome phosphorylation can be visualized on Western blots by an upward mobility shift of the phosphorylated protein. Prior studies have shown that cryptochrome phosphorylation in plant seedlings is altered as a function of the static magnetic field^3,19^, and is reduced at near null LLF (low -level fields). We therefore tested whether phosphorylation of the *Arabidopsis* cry1 receptor was also responsive to an applied weak 7 MHz RF magnetic field. This frequency was chosen as it had been previously reported to interfere both with bird navigation^13,20^ and with oxidative metabolic processes in mammalian cultured cells^6^ that have been postulated to involve flavoprotein radicals.

The experimental setup is described in detail in Methods. Briefly, a triaxial Helmholtz coil providing current along each of the three axes (x, y, z) was adjusted to set the static magnetic field parallel to the plane of growth of the seedlings, at 40 μT intensity to approximate the local geomagnetic field. Blue light LEDs were used to illuminate the sample. To generate the RF field, a single loop Helmholtz coil was placed around the sample on a rotating axis, such that an RF field could be set in a direction that was either at a parallel or a perpendicular angle to the static magnetic field (see Methods). The RF signal was 7 MHz at 2 μT_rms_. A low - level magnetic field (LLF or Low Level Field) of less than 200nT was generated by layering of sheets of μ-metal shielding around the sample (Methods). All experiments were performed in a dedicated darkroom with temperature at the position of the sample monitored in real time by computer throughout the course of the experiment.

Phosphorylation experiments were performed as described previously^3^. Four-day old dark-grown *Arabidopsis* seedlings on petri plates were illuminated for 90 minutes and simultaneously exposed to RF magnetic fields. These were applied either in parallel or in perpendicular to the geomagnetic field (see Methods). As the control condition, exposure was to the geomagnetic field alone (without applied RF). Finally, a series of sham experiments were conducted at each exposure condition to control for any background variation in the experimental setup (see Methods). Seedlings were then harvested and subjected to Western blot analysis with anti-Cry1 antibody to determine the cryptochrome protein upward mobility shift resulting from phosphorylation (Methods).

**Figure 1:**
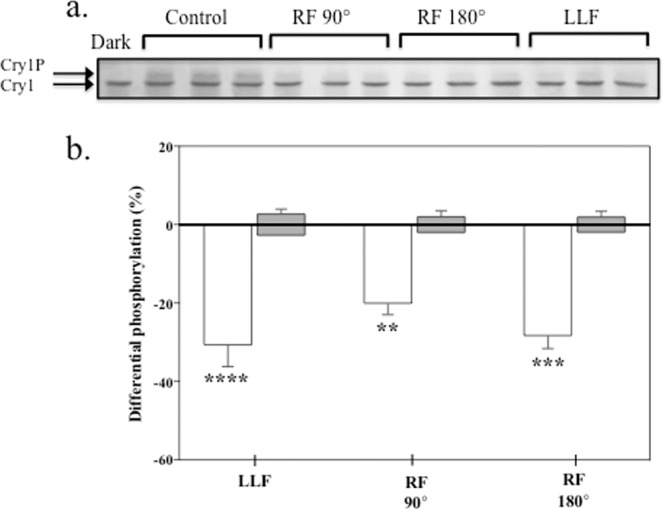
Phosphorylation of cry1 in response to 7 MHz RF fields. Four-day old dark-grown etiolated seedlings were subjected to 60 μmol m^−2^ s^−1^ blue light/dark cycles for 90 min as previously described^3^ and exposed to LLF (Low Level Field of less than 200nT) or to RF (7 MHz RadioFrequency field) applied either perpendicular (90°) or parallell (180°) to the static GMF (geomagnetic field) – see Fig. 5 in Methods. (**a**) Western blot of nonphosphorylated (Cry1) and phosphorylated (Cry1p) of bands detected by anti-cry1 antibody; triplicate samples were run for each exposure condition. Dark = seedlings before the onset of illumination; Control = seedlings were subjected to 60 μmol m^−2^ s^−1^ blue light/dark cycles for 90 min; RF and LLF exposure conditions are as described in the text. (**b**) Quantitation and statistical analysis of results from at least five independent experiments per exposure condition. Phosphorylation of cry1 is represented as the % difference between exposed seedlings as compared to seedlings maintained in the static GMF control condition. The results of sham experiments for each exposure condition (grey bars) represent differential phosphorylation between the seedlings in mock-treated LLF or RF (see Methods) and control GMF condition. The asterisks indicate a significance level of the differences: **p-value <0.01; ***p-value <0.001; ****p-value <0.0001. The effect of LLF (*p*-value <0.0001; N = 7), RF 90° (*p*-value = 0.008; N = 9) and RF 180° (*p*-value <0.001; N = 9); white bars; was in all cases to reduce cry1 phosphorylation. The sham treatments (N = 5) for each exposure condition yielded no significant difference compared to the control (GMF).

The results showed a significant (up to 24%) decline in response to blue light by cryptochrome in seedlings exposed to RF fields (Fig. 1). This was demonstrated by the reduced intensity of the upward-shifted, phosphorylated band in the Western blot under conditions of applied RF fields. Consistent with previous reports^19^, exposure to LLF conditions likewise caused a decrease in cryptochrome response (Fig. 1). Thus, an RF magnetic field has a similar effect on plant cryptochrome activation as does simply reducing the geomagnetic field to a LLF. Although our results by themselves are not proof that the same underlying mechanism is involved, it is nonetheless intriguing that an analogous effect has been documented in migratory birds^13,20^.

**Figure 2:**
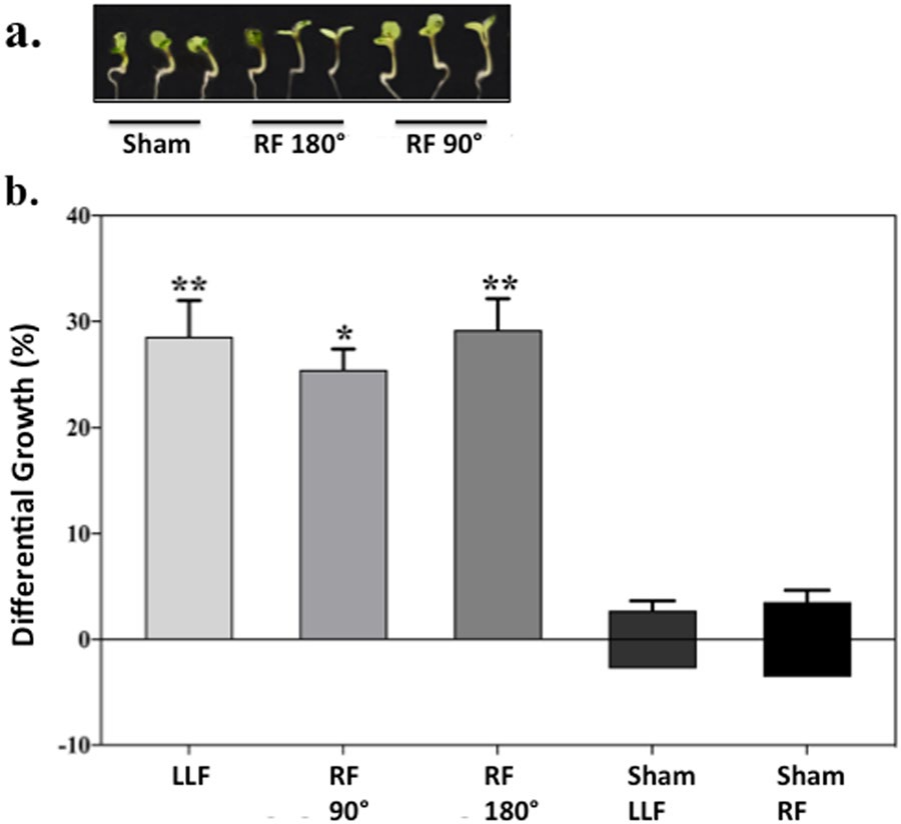
Effect of 7 MHz RF on *Arabidopsis* seedling hypocotyl growth inhibition. Seedlings were germinated and grown in 60 μmol m^−2^ s^−1^ blue light/dark cycles for 5 days as previously described^3^. During this time they were exposed to LLF (Low Level Field of less than 200nT) or to RF (7 MHz RadioFrequency field) applied either perpendicular (90°) or parallell (180°) to the static GMF (geomagnetic field). (**a**). Representative images of seedlings after exposure to RF. (**b**). Hypocotyl lengths averaged from 15 seedlings per exposure condition and compared to seedlings from the reference GMF (geomagnetic field) condition. The results were represented as % difference in hypocotyl length after exposure to LLF or RF as compared to the GMF (geomagnetic field) condition. As a control experiment, sham-exposed (mock treated) LLF and RF seedlings were compared to the reference GMF condition (grey bars). No significant difference between controls and sham-exposed controls was observed. Data are mean ± SE of five independent experiments. The asterisks indicate significance level of the differences: *p-value <0.05; ** *p*-value <0.01.

To further confirm these findings, two additional assays for cryptochrome biological activity were performed under conditions of RF exposure. These included *Arabidopsis* seedling hypocotyl growth inhibition and qPCR analysis of cryptochrome regulated gene expression, both of which have been reported sensitive to applied static magnetic fields^3,8^. For the plant growth experiments, seedling growth was monitored over 5 days under the identical illumination and magnetic field exposure conditions as for the cryptochrome phosphorylation experiments. Increased seedling growth is an indication of reduced cryptochrome biological activity, since cryptochrome mediates seedling growth inhibition under blue light^3^. The results of the plant growth assay showed that exposure to either parallel or perpendicular RF magnetic fields resulted in significantly increased hypocotyl length (up to 29%), and thereby reduced cryptochrome biological function, as compared to control seedlings (Fig. 2). Exposure to LLF conditions also caused increased hypocotyl length, indicating reduced cryptochrome response. Importantly, comparison of sham RF or sham LLF exposed seedlings to those at the reference geomagnetic field (GMF) condition yielded no statistical difference in phosphorylation.

**Figure 3:**
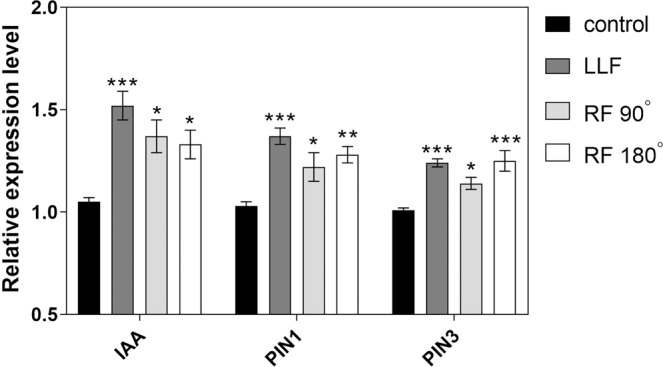
Effect of 7 MHz RF field on *Arabidopsis* gene expression. Four-day old dark-grown etiolated seedlings were subjected to 60 μmol m^−2^ s^−1^ blue light/dark cycles as previously described^3^ for 180 min and exposed to LLF (Low Level Field of less than 200nT) or to RF (7 MHz RadioFrequency field) applied either perpendicular (90°) or parallell (180°) to the static GMF (geomagnetic field). Expression levels of IAA, PIN1 and PIN3 genes of *Arabidopsis* was analysed by qPCR analysis as previously described^8^ – see also Methods. The results were presented as the relative expression level of the cryptochrome-regulated genes IAA, PIN1 and PIN3 after exposure to LLF, RF perpendicular, and RF parallel condition as compared to the reference GMF (geomagnetic field) condition. Data are mean ± SE of five independent experiments (N = 5). The asterisks indicate significance level of the differences: *p-value <0.1; ** *p*-value <0.01; ***p-value <0.001.

Expression analysis in response to RF magnetic fields was performed on *PIN1*, *PIN3*, and *AUX1* genes, which had previously been shown to be cryptochrome-regulated and responsive to static magnetic fields^8^. All three of these genes showed statistically significant change in expression in response to LLF exposure (Fig. 3), in this way replicating previously obtained results^8^. Upon exposure to RF fields, gene expression was altered to a similar degree as for LLF (Fig. 3). In summary, altered cryptochrome biological activity has been observed as a result of magnetic field exposure in three independent, unrelated biological assays; one of which (phosphorylation) provides a direct probe of the photoreceptor activation state.

## **Discussion**

**Figure 4:**
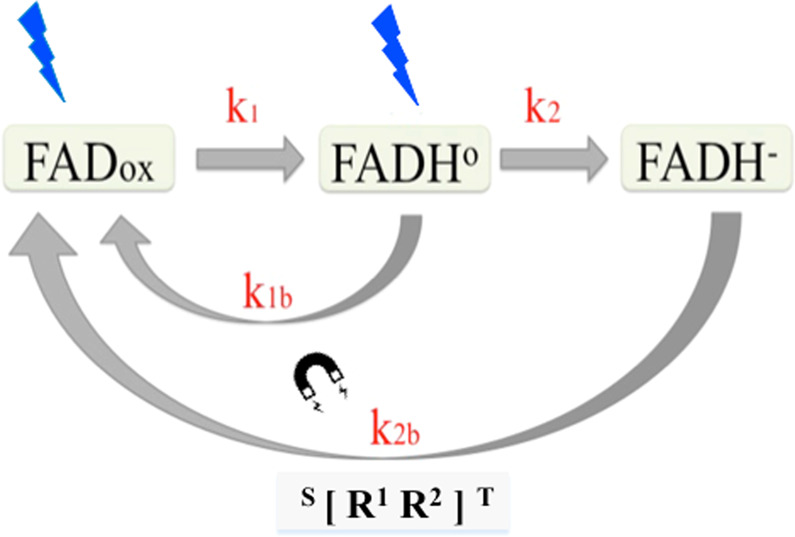
Model for effect of magnetic field on *Arabidopsis* cryptochrome. Please see ref. ^21^ and ref. ^22^ for full background. In the ground (dark adapted) state the FAD of cryptochrome exists in the fully oxidized redox state, generally referred to as FADox. Blue light illumination causes the flavin to be photoreduced to the neutral radical redox state, designated as FADH◦. The rate at which the flavin reduction reaction occurs at any given light intensity is defined by the rate constant k1, derived from the quantum yield for flavin photoreduction (shown by the blue arrow). The FADH° state is the biologically active signaling state. The radical flavin redox state (FADH◦) is further reduced to the fully reduced redox state (FADH-), with a rate constant k_2_. Flavin is reoxidized to the resting state (FADox) via mechanisms involving rate constants (k_1b_ and k_2b_) that are independent of light (black arrow). Since the FADH° redox state is the only biologically active state of the receptor, cryptochrome biological activity under a given illumination condition results from the equilibrium concentration of the active, FADH°, redox state as determined by the rate constants. *The effect of the magnetic field has been to specifically alter the rate constant k*_*2d*_^3,23^. A radical pair involving FADH° and possibly O_2_^o-^ (but see also discussion in ref. ^8^) formed in a triplet state has been suggested as mediating magnetic sensitivity. Given current uncertainty as to the identity of this radical pair we have labelled it $[R1R2{\boldsymbol{]}}$ Internal magnetic interactions that coherently interconvert radical-pair singlet and triplet spin states would then affect the rate (k_2d_) of product (FADox) formation. As a consequence, biological activity would be altered. This is because a change in k2d would change the equilibrium concentration of the active FADH° redox state during continuous blue light illumination.

Cryptochromes are proposed to be activated through flavin reduction in response to light, hereby conversion of oxidized flavin (FADox) to radical (FADH°) and reduced (FADH-) redox states triggers the conformational change leading to the biologically active form^21^ and ref. ^22^ here (see model in Fig. 4). Reduced flavin is reoxidized back to the inactive (FADox) redox state by a reaction that consumes molecular oxygen; this reaction has the potential to produce radical pair intermediates. Because only the FADH° redox state is correlated with biological activity, the response to light by cryprochrome is determined by the equilibrium concentration of FADH° under a given illumination condition. Intriguingly, prior studies have also shown that cryptochrome-dependent responsivity to magnetic fields occurs exclusively during the reoxidation phase of the photocycle^3,19,23^. Furthermore, a number of possible magnetically sensitive radical pairs may be formed in the course of reoxidation from fully reduced flavin (FADH-), suggesting a change in rate constant k_2b_ as the likely magnetically sensitive step.

We therefore calculated what magnitude of change in the rate constant k_2b_ could provide the observed magnetic field effect reported in Fig. 3. To model our experimental results in relation to the cryptochrome photocycle, we used a previously reported kinetic model which relates plant seedling growth to the average concentration of the FADH° (active) cry1 redox form^24^. This model uses previously deduced estimates of *in vivo* rate constants for cry1 flavin reduction/reoxidation steps (see Supplementary Information for details) in combination with experimental results from this study (Fig. 3). On the assumption that only k_2b_ (formed in the course of FADH- reoxidation) is modified by the magnetic field, we calculated that a modest increase in the rate constant of approximately 20% would result in the observed biological response to LLF. This is compatible with a possible radical pair mechanism^13,20^.

In conclusion, this study shows that RF magnetic fields alter the biological response characteristics of the *Arabidopsis* cryptochrome receptor itself, similarly to the effects of a near-null magnetic field. These results are consistent with the radical pair mechanism for magnetosensing and cannot be explained by an iron – based magnetosensor^13,20^, although we can not exclude that unrelated magnetosensing mechanisms exist in parallel^6,25,26^, or that cry may not be the direct RF receptor. Since cryptochromes are found in many organisms in the biological Kingdom including in humans, this study may lead to new biomedical applications developing RF signals to elicit desired cellular responses. Our results also may have more general implications for the capacity of living organisms to respond to man-made electromagnetic noise, by analogy with broad band RF^16^ which has been previously shown to disrupt orientation of birds.

**Figure 5:**
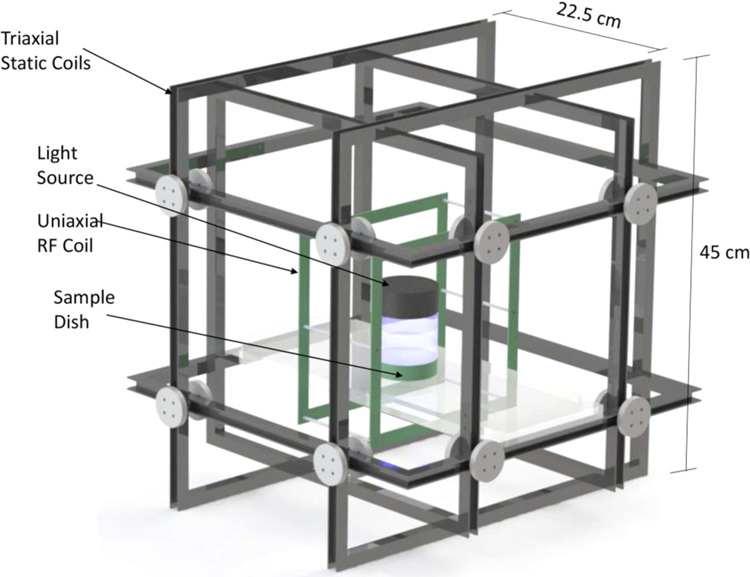
A diagram is shown that represents the experimental apparatus for magnetic field exposure system. Tri-dimensional representation of the tri-axial set used for controlling static and alternating electromagnetic fields. Square coil pairs in a Helmholtz configuration are geometrically aligned to control the static magnetic field (SMF) in the horizontal X-Y direction, and vertical (Z) direction. This diagram also depicts the placement of a square coil in Helmholtz configuration for the generation of RF magnetic fields^27^. For further details of exposure system and methodology, please see ref. ^27^, which used essentially identical apparatus.

## Materials and methods

### RF magnetic field exposure setup

The setup was essentially as previously described^27^. The initial static magnetic field (SMF) background inside the room varied from 25 to 60 μT as measured with a gauss meter (IDR-321, Integrity Design, VT, USA)^27^ in all 3 axes, and therefore required tri-axial compensation to establish a uniform pre-set SMF^27^ in the volumes designated for culture plates within the coils^27^. For these experiments, two tri-axial sets of square coils were constructed in a Helmholtz configuration^27^. The first set allowed for the simultaneous exposure of three 100 mm petri dishes as a control to a SMF of 40 μT^27^. The second set served to expose cells to a SMF of 40 μT and to either parallel/perpendicular applied weak 7 MHz magnetic fields^27^. In both cases, the 40 μT SMF was oriented perpendicular to the plane of growth of the plants^27^. The experimental exposure included both groups placed within separate tri-axial coils containing a single Helmholtz loop RF loop. The RF coil was not energized for the control SMF and was energized for the RF group^27^.

Each square coil (45 cm each side) consisted of 20 turns of 22 AWG enamel-coated copper wire^27^. Each pair of square coils was axially aligned and separated by 22.5 cm in order to achieve the Helmholtz configuration^27^. Each pair of coils in the Helmholtz configuration was individually driven by a power supply^27^. Resistive circuitry was fed in a twisted pair in order to achieve the necessary compensatory SMF in the desired direction^27^. The SMFs were adjusted accordingly at the isocenter of each tri-axial set as measured by a gauss meter for each axis^27^. A 1-turn square coil (12.5 cm side) in Helmholtz configuration was built inside one of the tri-axial sets in order to superimpose magnetic fields in the RF band also with 22 AWG enamel-coated copper wires^27^. The geometric center of this RF coil was aligned with that of the triaxial set used for SMF compensation^27^. A function generator (HP33120A, Hewlett-Packard, Palo Alto, CA) established the 7 MHz magnetic signal, and the magnitude recorded in the culture-designated volume was 2±0.5 μT (RMS) after power amplification^27^. The RF signal was measured with a circular search probe sensor composed of 5 turns of 22 AWG enamel-coated copper wires, 1.5 cm in radius, which were connected directly to an oscilloscope via a twisted pair feeding a coaxial cable^27^.

The background time-varying magnetic field was measured at the center of the tri-axial sets while inside the room in the location where the experiment was to be performed with a gauss meter (IDR-210, Integrity Design, VT) in all 3 axes^27^. The measurements performed resembled previous observations, where the dominant spectral magnitude was recorded at 50 Hz and was below 2 μT for all cases^27^. The temperature was maintained at 23 °C and verified throughout the course of the experiment by computer monitoring via a themocouple placed at the position of the sample^27^. The environmental parameter variance was minimal during the experiments^27^. The dark room was utilized exclusively for these experiments and were not opened for the duration of the exposures^27^.

### Near-null magnetic field Exposure System

The near-null magnetic field (LLF) was produced by a double layer μ-metal cylinder. The inner layer was 11.5 cm diameter, and the outer layer was 16 cm diameter, 30 cm of height. We measured the static magnetic field (SMF) at the center of cylinder, which was the position for *Arabidopsis* plate and the SMF intensity was lower than 200nT. The sham LLF was produced by a Helmholtz coil placed within the cylinder. Each coil consisted of 20 windings of 1 mm-diameter copper wire around a^3^ plastic circular frame (10 cm diameter, at a separation of 10 cm between coils) at the center^3^ of inner μ-metal cylinder (described in ref. ^3^). The current was provided to the coils to generate 40 µT static MF, which was local GMF for the sham-control experiments. The temperature was maintained at 23 °C and verified throughout the course of the experiment by computer monitoring via a themocouple placed at the position of the sample.

### Plant materials and growth conditions

*Arabidopsis thaliana phyA phyB* mutants were used for cry1 phosphorylation and qPCR gene expression experiments, *Arabidopsis thaliana* transgenic over-expressing cry1 seedlings were used in hypocotyl growth tests as previously described^3^. Seeds were sterilized by incubation with 25% bleach for 30 min, washed 3X with sterile water, and plated on 5 mm diameter petri dishes containing 2% (W/V) sucrose, 0.5X MS salts pH 6.0 (MP Biomedicals, INC, Illkirch, France) and 0.9% (W/V) agar. Plates were maintained at 4 °C in the darkness for 48 hours, then illuminated with red light (633 nm) at 23 °C for 24 hours. For phosphorylation and qPCR assay experiments, seedlings were returned to darkness at 23 °C for 4 days. Seedlings for hypocotyl growth assays were transferred after germination to blue light test conditions for 5 days growth. Between 100 and 200 seeds per plate were used for the phosphorylation assay; 15 seeds per plate were measured for the hypocotyl growth assay. Details are as previously described in previous refs. ^3,22^.

### Blue-light exposure system

Please see^3^ for complete details. Blue light was produced by LEDs with peak wavelength of 447 nm (Quadica Developments Inc., Alberta, Canada) mounted 4.5 cm above the seedlings at the center of the exposure coil (see above). The LEDs were controlled by custom built automated programmable switches (see ref. ^3^. for details) to provide alternating 5 min blue light / 10 min dark pulsed illumination conditions. The photon fluence of blue-light intensity for the experiment was measured by Quantum light meter (LI-185B, LI-COR, Inc., USA).

### Phosphorylation assay

Details of this procedure are taken from^3,22^. The phosphorylation assay was performed as described previously^3^. 4-day old dark-grown *Arabidopsis* seedlings were exposed to treatment conditions as follows: 7 MHz RF oriented perpendicular or parallel to the Static Magnetic Field; or LLF (low level field). The control condition was the geomagnetic field at 40 μT. Illumination at all exposure conditions was identical, consisting of repeated pulses of blue-light at 60 µmolm-^2^s^−1^ for 5 min followed by 10 min darkness. This cycle was repeated 6 times for a total time of 90 min. The temperature was maintained at 23 °C and verified throughout the course of the experiment by computer monitoring via a themocouple placed at the position of the sample. Seedlings were then quick-frozen in liquid nitrogen and total protein extracted and assayed for the presence of the cryptochrome phosphorylated band by Western blotting as previously described^3^.

### Experimental design and controls

The experimental design to demonstrate the effect of RF and LLF on cry1 phosphorylation was as previously described^3^. To eliminate any possibility of artifact between experiment (test) and control comparisons, the experiments were performed in duplicate sets of at least five independent trials each. The two experimental groups were designated *treatment*, and *control group*, respectively, each of which were replicated in at least five independent biological repeats.

To determine the extent of phosphorylation, the intensity of the upper, phosphorylated band from each lane of the Western blot (cry1(Pi)) (see example gel Fig. 1, upper panel) was determined using imaging software ImageJ and expressed as a percentage of the intensity of the total cry1 protein (sum of phosphorylated plus unphosphorylated cry1) in the same lane. The formula for obtaining the extent of Cry phosphorylation is thereby [cry1(Pi)]/ [cry1 (total)] x 100 yielding the percentage of phosphorylated cryptochrome per lane. Three triplicate lanes per individual experimental condition were averaged to yield the percentage of phosphorylation in one harvested sample.

For the *treatment group*, the mean cry1 phosphorylation value of each exposure condition (RF parallel, RF perpendicular, LLF) was compared to the mean cry1 phosphorylation in the reference static 40 μT condition designated as Geomagnetic Field (GMF). Values were expressed as the percent difference between phosphorylation in exposed and reference samples (see Fig. 1). For the *control group*, we compared the mean of cry1 phosphorylation in sham - exposed samples to those of cry1 phosphorylation at the reference GMF condition; (RF perpendicular sham, RF parallel sham, or LLF sham vs. GMF reference). In this *control group*, the experimental setup, position of samples, illumination of samples, etc. were identical to that of the *test experimental group* except that no RF or LLF was applied *in the test* conditions (ie a mock ‘test’ group).

We then performed statistical analysis to determine the percentage difference of cry1 phosphorylation within the *treatment group* (RF perpendicular, N = 7; RF parallel, N = 9; LLF, N = 7) and within the *control group* (N = 5 for sham - exposed RF perpendicular, RF parallel, and LLF) (see below).

### Hypocotyl growth experiments

Seedling growth experiments were performed as in^3^. For each experimental determination, 15 seedlings were measured. All analysis was performed double blind, in that the person performing the measurements did not know under which condition the plates had been grown. For the *treatment group*, the mean value of hypocotyl length from each treatment condition (RF perpendicular, RF parallel, LLF) was compared to the mean value of hypocotyl length in seedlings exposed to the reference GMF condition. For the *control group*, comparisons were made between sham-treated seedlings and the reference GMF condition. Five replicate biological repeats were performed for all experiments in both the *treatment* and *control* groups (N = 5).

For statistical analysis, the significance of differential growth from each condition in the *treatment group* (N = 5; +RF perpendicular vs. GMF, + RF parallel vs. GMF, + LLF vs. GMF) and from each condition of the *control group* (N = 5; -RF perpendicular vs. GMF, -RF parallel vs. GMF, -LLF vs. GMF) was calculated.

### Quantitative RT-PCR analysis of altered gene expression

QPCR analysis was performed as described^8^. Dark-grown etiolated *Arabidopsis* seedlings were plated as for the phosphorylation assay, then illuminated at 60 µmolm^−2^s^−1^ blue-light for 5 min followed by 10 min darkness. This cycle was repeated for a total time of 3 h, under the designated electromagnetic exposure conditions (RF, LLF, or the reference GMF). After quick-freezing in liquid nitrogen, total RNA was extracted using the Total RNA Miniprep Kit (New England Biolabs) and cDNA was prepared from 1 mg total RNA using SuperScript first-strand synthesis system (Thermo Fisher Scientific). Quantitative RT-PCR was performed using Luna qPCR master mix (New England Biolabs). *Arabidopsis* GADPH was used as the reference gene^28,29^. Five biological replicates were performed for each analysis (N = 5). Primers used for gene expression analysis are listed in Table 1.

**Table 1:** List of primers used in the current study.

Gene	Primer name	Primer sequence (5’-3’)	Primer Reference
*IAA*	IAA-Fw	TGGTCGGTGATGTTCCAT	^15^
	IAA-Rev	CGGATCCTTTCATGATTCTG	
*PIN1*	PIN1-Fw	AACCACCACGCCGAATTACTC	^30^
	PIN1-Rev	CACCGTCCGTTGCCAATACT	
*PIN3*	PIN3-Fw	TCTTATCCGGCTCCGAAT	^15^
	PIN3-Rev	GAAGCTCCTTGGCGTCAT	
*GADPH*	GADPH-Fw	TTGGTGACAACAGGTCAAGCA	^28^
	GADPH-Rev	AAACTTGTCGCTCAATGCAATC	

Data and statistical analysis.

For statistical analysis, all data were analyzed by using GraphPad Prism version 7.00 for Windows (GraphPad Software, La Jolla California, USA). Data were analyzed for normality with the Shapiro-Wilk test and Homogeneity of Variances with the Brown-Forsythe test. Results are expressed as means ± standard error of the mean (SEM). The difference between RF or LLF exposed and reference (GMF) samples were compared by using One-way ANOVA followed by Bonferroni’s multiple comparisons test. Differences were considered statistically significant with a *p*-value < 0.05 (*), < 0.01 (**), < 0.001 (***) and <0.0001 (****).

## Supplementary information

Supplementary information 1

Supplementary information 2
