# Supplementary material for: Arabidopsis cryptochrome is responsive to Radiofrequency (RF) electromagnetic fields
Source: Sci Rep. 2020 Jul 9;10:11260. doi: 10.1038/s41598-020-67165-5 (PMC7347919; doi:10.1038/s41598-020-67165-5)

Supplementary Information

***Arabidopsis* cryptochrome is responsive to Radiofrequency (RF) electromagnetic fields.**

**Authors:** Maria Albaqami ^1,2#^, Merfat Hammad ^1,2#^, Marootpong Pooam ^1#^, Maria Procopio ^3^,Mahyar Sameti^2^, Thorsten Ritz ^4^, Margaret Ahmad ^1,5*^ and Carlos F Martino ^2^.

**Affiliations:**

^1^Sorbonne Universités – UPMC Paris 6 – CNRS, UMR8256 - IBPS, Photobiology Research Group, 7 Quai St. Bernard, 75005 Paris France.

^2^Department of Biomedical and Chemical Engineering and Science, Florida Institute of Technology, 150 W University Blvd, Melbourne, Fl 32901 U.S.A.

^3^Department of Biophysics, Johns Hopkins University, 3400 N. Charles Street, Baltimore, MD 21218 U.S.A.

^4^Department of Physics and Astronomy, University of California at Irvine, U.S.A.

^5^Xavier University, 3800 Victory Parkway, Cincinnati, Ohio 45207, U.S.A.

*Correspondence to: [margaret.ahmad@upmc.fr](mailto:margaret.ahmad@upmc.fr)

# These authors contributed equally

1. **Interpretation of magnetic field effects via kinetic modeling**

**Introduction:**

The purpose of this section is to estimate the effect a magnetic field could have on Cry1 to obtain our observed decrease in biological activity under low level static magnetic field (LLF) conditions (Fig. 3). Specifically, we calculate the reaction rate change in the Cry flavin redox cycle that would be consistent with a corresponding decrease in the biologically active (FADH°) signaling state.

We use a kinetic modeling approach that has been previously described for the Cry1 photocycle in response to light in *Arabidopsis* (1). In this model, the Cry photocycle is conceived as shown in Fig.4 in the main text: in the resting (dark) state, Cry occurs with flavin in the oxidized (FADox) redox form which is biologically inactive. Illumination triggers formation of the biologically active radical (FADH°) redox form (rate constant k_1_). FADH° in turn can be further reduced to the biologically inactive (FADH-) redox form (rate constant k_2_). Reoxidation occurs either from reduced (FADH-) (k_2b_) or radical (FADH°)(k_1b_) flavin to restore the FADox resting state (2). The model uses as assumptions the following previously reported estimates of k_1_=0.4 s^-1^ and k_2_=0.04 s^-1^ (1, 2). Reduced reaction intermediates FADH° and FADH- are relatively long-lived. In our model reoxidation k values are assumed as k_1b_ =$0.0019 \pm$ 0.0001 sec^-1^ (see ref. 3, which reports an error of 5.6% in Cry1 reoxidation rate *in vivo*). k_2b_ has been reported as 2.5x k_1b_ *in vitro* (2). For the purposes of these calculations we make the assumption that the same relationship holds *in vivo*, providing a k_2b_ of 0.0047 $\pm$ 0.00026 sec.^-1^ Our final assumption is that the magnetic field effect (MFE) occurs solely during the light-independent reoxidation step of the flavin photocycle (4), and that only the reoxidation reaction from FADH- ( k_2b_ ) could generate possible magnetically sensitive radical pairs (2).

Therefore, our approach is to calculate the magnitude of the change in rate constant k_2b_ that would be sufficient to account for the magnetic field effect observed in this study.


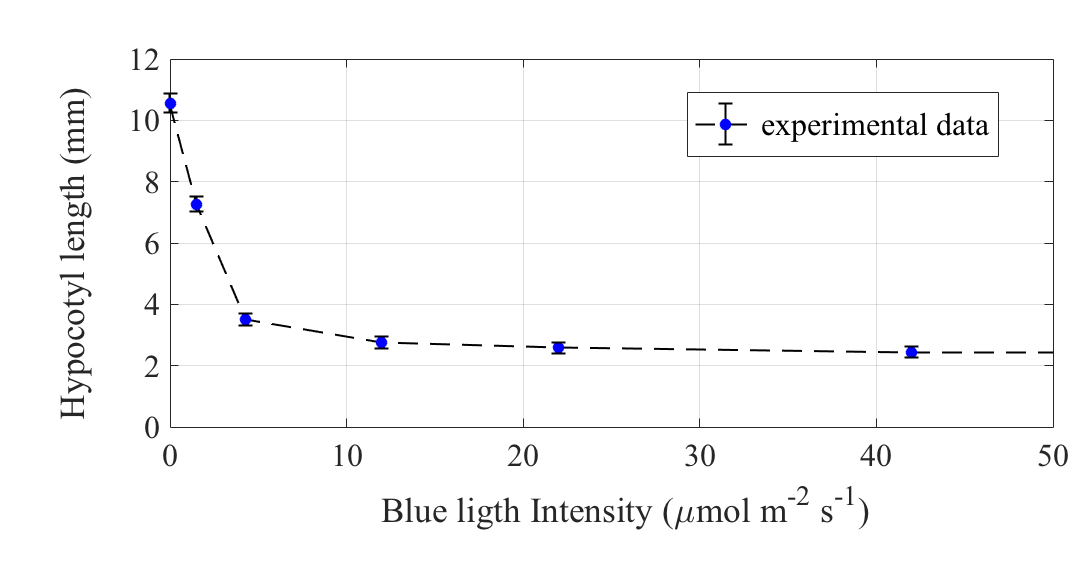


**Figure S1**. Hypocotyl growth inhibition under pulsed light conditions. Cry1 overexpressing seedlings were grown at increasing intensities of blue light illumination under repetitive cycles of 5min light, 10min dark pulses for five days. These are the same pulsed illumination conditions as were used for the magnetic field experiments (Fig. 1 – 3). Maximal growth inhibition occurred at less than 10 μmoles m^-2^sec^-1^. Error bars are SE.

**2. Modelling of Cryptochrome Photocycle**

**Underlying Assumptions**

FADH° is the biologically active redox state.

FADH^-^ reoxidation forms radical-pairs: the k_2b_ rate constant is altered by magnetic fields

k_1_=0.4 s^-1^

k_2_=0.04 s^-1^

k_1b_ =$0.0019$sec^-1^

k_2b_ = 0.0047 sec.^-1^

**Three states Kinetic Model**

We use a three states kinetic model, reported in Eq. S1, to calculate the changes in the k_2b_ re-oxidation rate that could account for magnetic field effects on the *Cry1* photocycle as previously described (see ref. 1).

$$\left\{ \begin{aligned} \frac{d\left[ {FAD}_{ox} \right]}{dt}={-k}_{1}\left[ {FAD}_{ox} \right]+k_{1b}\left[ {FADH}^{o} \right]+k_{2b}\left[ {FADH}^{-} \right] \\ \frac{d\left[ {FADH}^{o} \right]}{dt}=k_{1}\left[ {FAD}_{ox} \right]-(k_{2}{+k}_{1b}\left[ {FADH}^{o} \right] \\ \frac{d\left[ {FADH}^{-} \right]}{dt}=k_{2}\left[ {FADH}^{o} \right]-k_{2b}\left[ {FADH}^{-} \right] \end{aligned} \right. (S1 )$$

We set the initial normalized concentration of *Cry1* at time t=0 to [FAD_ox_]=1. At any given later time t, ${[FAD}_{ox}]\left( t \right)+{[FADH}^{o}]\left( t \right)+\left[ {FADH}^{-} \right]\left( t \right)=$1.


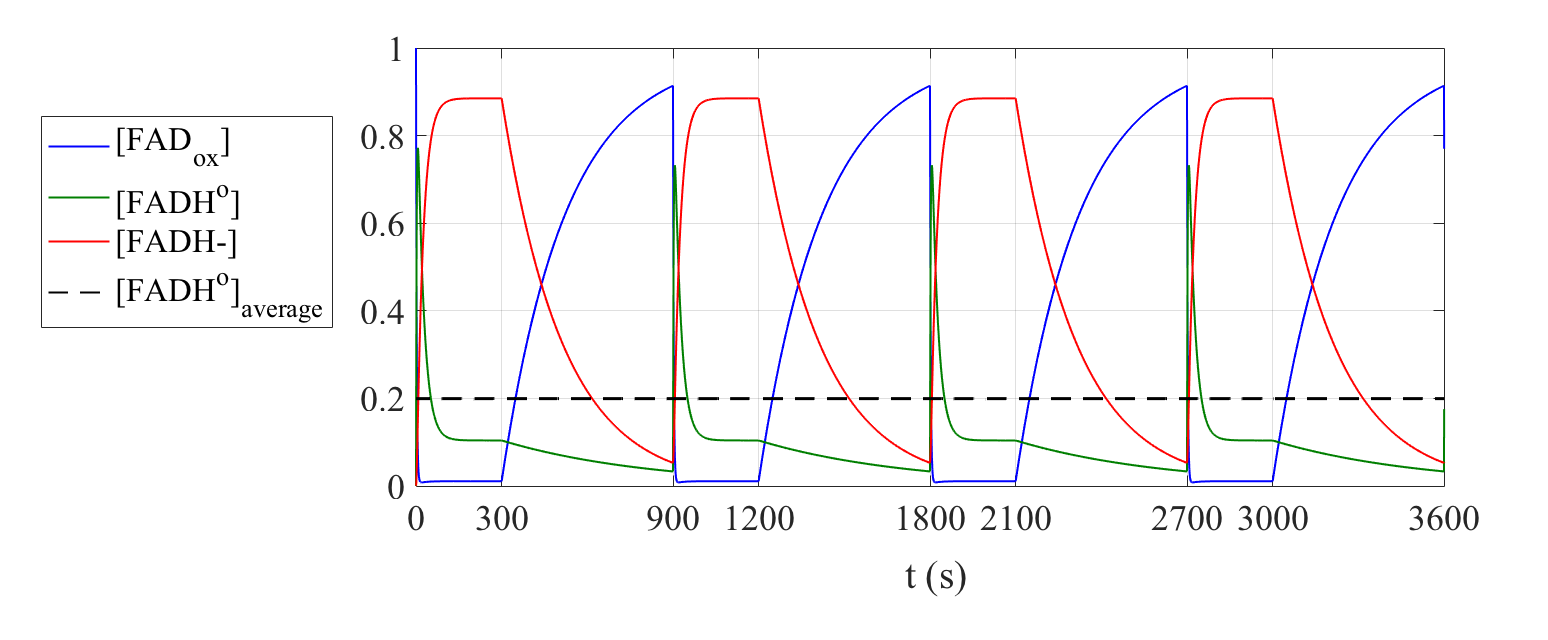


Figure S2. Calculated concentrations of Cry redox state intermediates during pulsed illumination. FADox (solid blue), FADH° (solid green) and FADH- (solid red) intermediate states are shown to cycle in accordance with light/dark cycles. We only show here normalized concentrations under a few cycles, which are stable over all 5 days, based on the 3 states model. 1 cycle is 5 min blue light, followed by 10 min dark. Dotted black line indicates averaged FADH° concentration over the light/dark period,

**Setting the *Control* rate constants for the three states model**

The control condition is at 40μT, under illumination conditions of 60 μmoles m^-2^sec^-1^, well above light saturation for biological activity (see Figure S1). This allows that after the 5 min light exposure we may consider ${[FAD}_{ox}]=0$ i.e. that it is completely depleted, and using the above given assumptions for k_2_, provides an approximate ${[FADH-}]=0$.9. By numerically solving Eq. S1, we calculate the normalized concentration of ${[FAD}_{ox}] (blue),$[*FADH^o^*] (*green*) and $\left[ {FADH}^{-} \right] \left( red \right)$as a function of time, under the experimental blue light conditions of 5 minutes light followed by 10 minutes darkness over a period of time of 5 days (see Figure S2). The calculated average [FADH^o^] over time during 5 days cycling is shown as $\left[ {FADH}^{o} \right]_{average}= 0.2 \pm0.0065$ (dotted black line in Figure S2). The error was calculated by considering the errors of k_1b_ and k_2b_ reported in ref.3. The hypocotyl length of seedlings in the control condition, and the corresponding average [FADH^o^] to provide this biological response are reported in Figure S3. We use these forward and back reactions rates, as a reference to calculate changes during LLF.

**Calculated changes in re-oxidation rate k_2b_ under LLF**

During the LLF exposure, the hypocotyl length increased by about 28%, corresponding to a 10% decrease in $\left[ {FADH}^{o} \right]_{average}$ (taken from analysis of relationship between hypocotyl growth inhibition and *in vivo* FADH° concentration in reference 1). Thus under LLF, the estimated ${[{FADH}^{o}]}_{average}^{LLF}=0.18$. We further assume that the magnetic field exclusively affects the reoxidation rate k_2b_ (back reaction from $\left[ {FADH}^{-} \right]$ to ${[FAD}_{ox}]$ . To find the effect of the magnetic field on this rate, we calculate k2b for the LLF hypocotyl growth condition by solving Eq. S1 for the change in k_2b_ value that gives rise to the new average ${[{FADH}^{o}]}_{average}^{LLF}= 0.18$. We find a new $k_{2b}^{LLF}= 0.0038.$ By considering an error of 5.6% in k_2b_ we find $k_{2b}^{LLF}= 0.0038 \pm0.00021$ and ${[{FADH}^{o}]}_{average}^{LLF}= 0.18 \pm0.005$.

Thus, while under the control local field condition, the $\boldsymbol{k}_{\boldsymbol{2}\boldsymbol{b}}\boldsymbol{=}\mathbf{0.0047}\boldsymbol{\pm0.00026}\boldsymbol{s}^{\boldsymbol{-1}}$, under the LLF test condition the reoxidation rate k_2b_ would need to change to $\boldsymbol{k}_{\boldsymbol{2}\boldsymbol{b}}^{\boldsymbol{LLF}}\boldsymbol{= 0.0038 \pm0.00021}\boldsymbol{s}^{\boldsymbol{-1}}$ to provide the observed biological phenotype using our given assumptions, which means it should decrease by about 20% (19.2%).


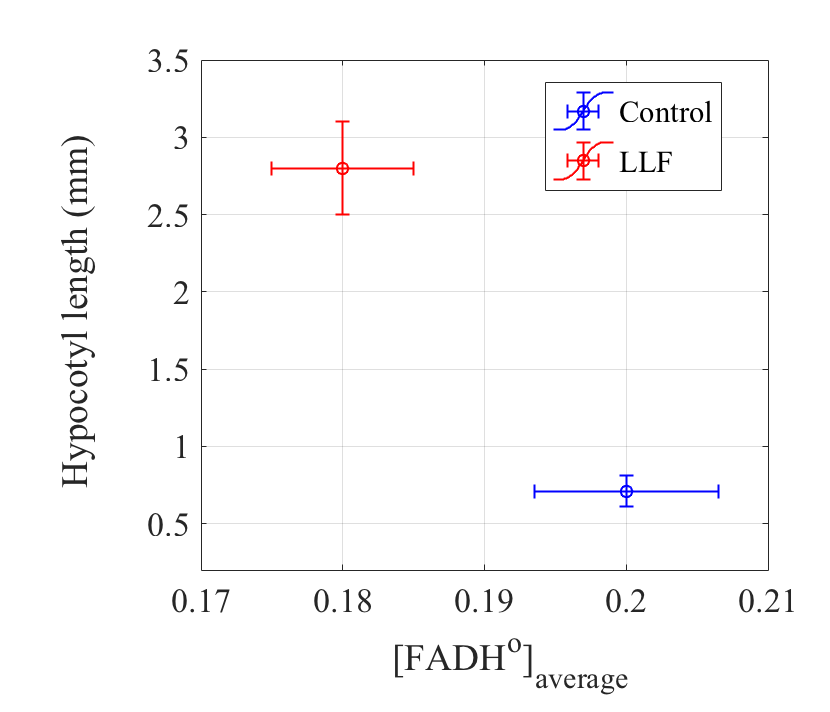


Figure S3. Change in FADH° concentration consistent with LLF exposure. Hypocotyl length in mm is plotted as a function of the calculated average concentration of [FADH^o^] for the control (blue) and test LLF (red) condition phenotype. Details of [FADH^o^] calculations are described in the text and in ref. 1. Error bars for *in vivo* measurements are taken from reference 3.

**Discussion.**

The goal of this simulation is to discuss our experimental results with respect to the Radical Pair mechanism of magnetoreception. The Radical Pair model predicts that electromagnetic fields could cause small alterations in biochemical reaction rates involving unpaired radical intermediates. Here we calculate that an increase of around 20% in the rate for flavin reoxidation (from FADH- to FADox) should result in the observed decrease in plant biological activity under LLF conditions. This change is, firstly, on an order of magnitude consistent with the RP hypothesis. Secondly, the affected reaction is one that is known to produce radical pairs (2), and which is under consideration with respect to the Radical Pair hypothesis (see eg. 5, 6).

It should nevertheless be emphasized that our simulation is based on a number of assumptions that can not be directly verified. For instance rate constants for reoxidation *in vivo* are obtained by indirect means (from refence 3) or estimated from rate constants determined *in vitro* (eg. from reference 2). Furthermore, the magnitude of the biological phenotype being assessed (seedling growth) may not be directly proportional to the primary photochemical steps modified by the magnetic field. This is because plant growth involves literally dozens of intermediate steps from light perception to phenotype that may modulate the response independently of the magnetic field. For all these reasons, the error bars shown on Figure 3 are likely to be considerably underestimated. Nonetheless, we consider truly gross errors, such as those approaching an order of magnitude, to be extremely unlikely given the quantitative nature of the biological response and physical constraints of the underlying flavin photochemistry.

As a result, certain valuable conclusions can be drawn from this analysis. Our model allows us for the first time to discuss a known biochemical reaction in a biological receptor in terms of a magnetically sensitive *in vivo* response. This reaction (redox transition from FADH° to FADox) moreover makes certain clear predictions concerning the possible magnetically sensitive radical pairs involved (see ref. 5, 6 and discussions therein). We have furthermore been able to predict the approximate magnitude of the change in reaction rate that would be required for our experimentally determined response to LLF. Although still awaiting more direct experimental confirmation, this simulation thereby places the cryptochrome-dependent response to LLF squarely within the parameters of a possible Radical Pair mechanism.

**Bibliography**

1. Procopio, M, J Link, D Engle, J Witczak, T Ritz, and M Ahmad. "Kinetic modeling of the Arabidopsis cryptochrome photocycle: FADHo accumulation correlates with biological activity." *Front. Plant Sci* 7 (2016): 888.

2. Mueller P, Ahmad M. 2011 Light-activated cryptochrome reacts with molecular oxygen to form a flavin-superoxide radical pair consistent with magnetoreception. *J. Biol. Chem*. 286, 21 033–21 040.

3. Herbel, Vera and Orth, Christian and Wenzel, Ringo and Ahmad, Margaret and Bittl, Robert and Batschauer, Alfred. "Lifetimes of Arabidopsis cryptochrome signaling states in vivo." *The Plant Journal* 4 , no. 0960-7412 (2013): 583-592.

4. Pooam M, Arthaut LD, Burdick D, Link J, Martino CF, Ahmad M. 2019 Feb, and 249(2):319-332. "Magnetic sensitivity mediated by the Arabidopsis blue-light receptor cryptochrome occurs during flavin reoxidation in the dark." *Planta.* 249, no. 2 (2019): 319-332.

5. Mondal P, Huix-Rotllant M. Theoretical insights into the formation and stability of radical oxygen species in cryptochromes. *Phys. Chem. Chem. Phys*. DOI: 10.1039/c9cp00782b (2019)

6. Kattnig D. Radical-Pair-Based Magnetoreception Amplified by Radical Scavenging: Resilience to Spin Relaxation. J Phys Chem B. 2017, 121, 44, 10215-10227

Western Blot Images


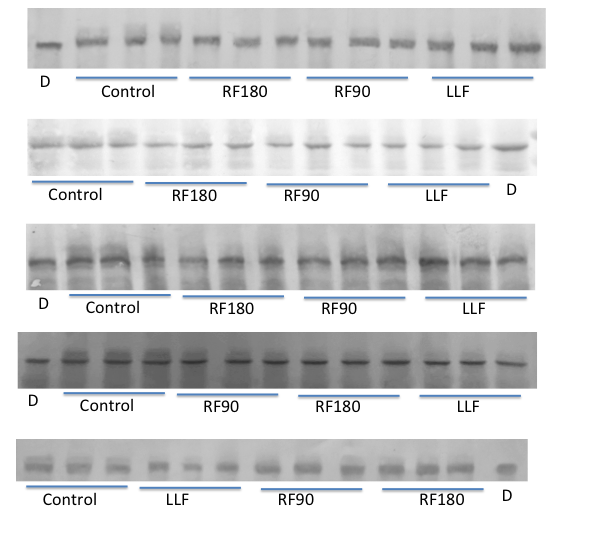


Western Blot Raw Image (Fig.2)


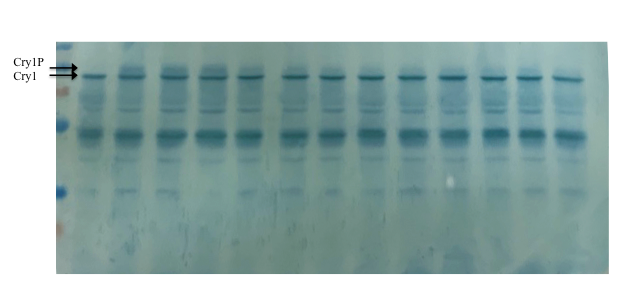

Supplement: Supplementary file 1 — Supplementary information 1 [file 41598_2020_67165_MOESM1_ESM.docx]
